# *N*-(2-Fluoro­phen­yl)-5-[(4-meth­oxy­phen­yl)amino­meth­yl]-6-methyl-2-phenyl­pyrimidin-4-amine

**DOI:** 10.1107/S1600536811045028

**Published:** 2011-11-05

**Authors:** Jerzy Cieplik, Janusz Pluta, Iwona Bryndal, Tadeusz Lis

**Affiliations:** a Department of Organic Chemistry, Medical Academy, 9 Grodzka St, 50-137 Wrocław, Poland; b Faculty of Chemistry, University of Wrocław, 14 Joliot-Curie St, 50-383 Wrocław, Poland; c Department of Bioorganic Chemistry, Faculty of Engineering and Economics, Wrocław University of Economics, 118/120 Komandorska St, 53-345 Wrocław, Poland

## Abstract

The conformation of the title mol­ecule, C_25_H_23_FN_4_O, is mainly determined by an intra­molecular N—H⋯N hydrogen bond closing a six-membered ring and the dihedral angles between the pyrimidine ring and the three benzene rings which are 12.8 (2), 12.0 (2) and 86.1 (2)°. An intra­molecular N—H⋯F inter­action also occurs. The crystal stucture is stabilized by weak C—H⋯O and C—H⋯π inter­actions. An inter­molecular N—H⋯N inter­action is also observed.

## Related literature

For anti­bacterial activity of 6-methyl-2-phenyl-5-substituted pyrimidine derivatives, see: Cieplik *et al.* (1995 ▶, 2003 ▶, 2008 ▶); Pluta *et al.* (1996 ▶). For related structures, see: Cieplik *et al.* (2006 ▶).
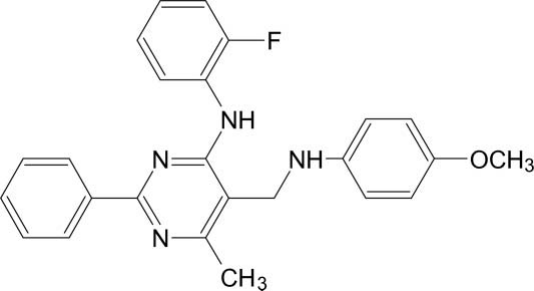


## Experimental

### 

#### Crystal data

C_25_H_23_FN_4_O
*M* *_r_* = 414.47
Monoclinic, 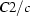

*a* = 27.075 (11) Å
*b* = 8.922 (4) Å
*c* = 22.983 (10) Å
β = 132.22 (5)°
*V* = 4112 (3) Å^3^
*Z* = 8
Mo *K*α radiation
μ = 0.09 mm^−1^
*T* = 85 K
0.53 × 0.17 × 0.14 mm

#### Data collection

Oxford Diffraction Xcalibur PX κ-geometry diffractometer
28521 measured reflections
10469 independent reflections
7196 reflections with *I* > 2σ(*I*)
*R*_int_ = 0.025

#### Refinement

*R*[*F* ^2^ > 2σ(*F* ^2^)] = 0.044
*wR*(*F* ^2^) = 0.121
*S* = 1.01
10469 reflections
288 parameters
H atoms treated by a mixture of independent and constrained refinement
Δρ_max_ = 0.61 e Å^−3^
Δρ_min_ = −0.22 e Å^−3^

### 

Data collection: *CrysAlis CCD* (Oxford Diffraction, 2007 ▶); cell refinement: *CrysAlis RED* (Oxford Diffraction, 2007 ▶); data reduction: *CrysAlis RED*; program(s) used to solve structure: *SHELXS97* (Sheldrick, 2008 ▶); program(s) used to refine structure: *SHELXL97* (Sheldrick, 2008 ▶); molecular graphics: *XP* in *SHELXTL* (Sheldrick, 2008 ▶); software used to prepare material for publication: *SHELXL97*.

## Supplementary Material

Crystal structure: contains datablock(s) I, global. DOI: 10.1107/S1600536811045028/gk2418sup1.cif

Structure factors: contains datablock(s) I. DOI: 10.1107/S1600536811045028/gk2418Isup2.hkl

Supplementary material file. DOI: 10.1107/S1600536811045028/gk2418Isup3.cml

Additional supplementary materials:  crystallographic information; 3D view; checkCIF report

## Figures and Tables

**Table 1:** Hydrogen-bond geometry (Å, °) *Cg*1, *Cg*2 and *Cg*3 are the centroids of the N1–C6, C41–C46 and C21–C26 rings, respectively.

*D*—H⋯*A*	*D*—H	H⋯*A*	*D*⋯*A*	*D*—H⋯*A*
N4—H4⋯N5	0.88 (1)	2.07 (1)	2.794 (2)	139 (1)
N4—H4⋯F4	0.88 (1)	2.19 (1)	2.633 (1)	111 (1)
C61—H611⋯O5^i^	0.98	2.49	3.426 (2)	159
C61—H612⋯*Cg*1^ii^	0.98	2.69	3.381 (3)	128
C61—H613⋯*Cg*2^iii^	0.98	2.73	3.657 (3)	159
C53—H53⋯*Cg*3^ii^	0.95	2.74	3.634 (3)	156
N5—H5⋯N1^iii^	0.90 (1)	2.76 (1)	3.541 (2)	145 (1)

Symmetry codes: (i) 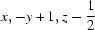; (ii) 
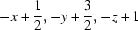
; (iii) 
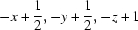
.

**Fig. 1.**
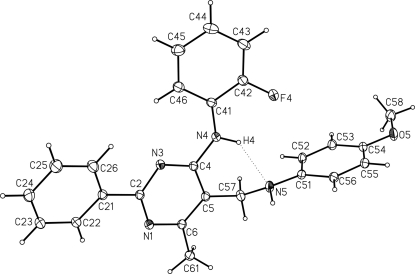
Molecular structure of the title compound with displacement ellipsoids drawn at the 50% probability level and H atoms shown as small spheres of arbitrary radii. The dotted line indicates an intramolecular N—H···N hydrogen bond.

**Fig. 2.**
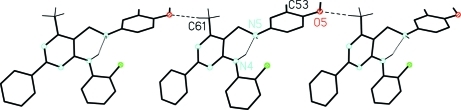
Part of the crystal structure viewed down the b axis showing the chain formed via C—H···O hydrogen bonds. Dotted and dashed lines indicate intra- and intermolecular hydrogen bonds, respectively. H atoms not involved in hydrogen bonding have been omitted for clarity.

**Fig. 3.**
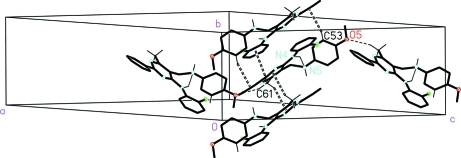
A packing diagram of title compound, showing C—H···π interactions. Intermolecular C—H···O interactions are shown with dashed lines, C—H···π interactions with double dashed lines and intramolecular N—H···N interactions with dotted lines. H atoms not involved in hydrogen bonding have been omitted for clarity.

## supplementary crystallographic information

### Comment

Pyrimidines are very important molecules in biology and have many applications in the areas of pharmaceuticals. 6-Methyl-2-phenyl-4-thio-5-pyrimidine carboxylic acid is a key intermediate for our synthesis of a wide range of various 6-methyl-2-phenyl-5-substituted pyrimidine derivatives (Cieplik *et al.*, 1995, 2003, 2008; Pluta *et al.*, 1996). Microbiological testing of a number of such pyrimidine derivatives, designed and synthesized as immunomodulating agents, showed them to possess antibacterial and antifungal activity (Cieplik *et al.*, 1995, 2003). In connection with these studies, we have described the structures of two polymorphic forms of *N*-(4-chlorophenyl)-5-[(4-chlorophenyl)aminomethyl]-6-methyl-2-phenylpyrimidin-4-amine (Cieplik *et al.*, 2006). Now, we have prepared a new compound of this class of derivatives, namely *N*-(2-fluorophenyl)-5-[(4-methoxyphenyl)aminomethyl]-6-methyl-2-phenylpyrimidin-4-amine and present its structure here. In the crystal, the orientation of the amine groups with respect to each other results from intramolecular N—H···N hydrogen bond between N4—H4 and N5, which closes six-membered ring. Conformation of the title molecule is best defined by the dihedral angles formed between the pyrimidine ring plane and the planes of the phenyl group attached to C2 and the benzene rings of the (2-fluorophenyl)amino or (4-methoxyphenyl)aminomethyl groups, attached to C4 or C5 of the pyrimidine ring. These dihedral angles are 12.8 (2)°, 12.0 (2)° and 86.1 (2)°, respectively. The twist of 12.8 (2)° of the phenyl group is similar to that found in the polymorphic forms of *N*-(4-chlorophenyl)-5-[(4-chlorophenyl)aminomethyl]-6-methyl-2-phenylpyrimidin-4-amine [5.2 (2)° and 6.4 (2)°; Cieplik *et al.*, 2006]. Additionally, the aromatic atom C41 of the (2-fluorophenyl)amino group is nearly coplanar with the pyrimidine ring. The C51 atom of (4-methoxyphenyl)aminomethyl group deviates from the pyrimidine ring plane by -1.01 (1) Å.

Contrary to polymorhic forms of *N*-(4-chlorophenyl)-5-[(4-chlorophenyl)aminomethyl]-6-methyl-2-phenylpyrimidin-4-amine (Cieplik *et al.*, 2006), the amine atom N5 does not participate in hydrogen bonding. The crystal structure of title compound is stabilized by weak C—H···O and C—H···π hydrogen bonds. In the crystal, the molecules are linked by C—H···O hydrogen bonds involving the methyl group C61 as a donor and O5 atom (*x*, -*y* + 1, *z* - 1/2) of the methoxy group as an acceptor. This interaction links the molecules into polymeric chains parallel to the *c* axis (Fig. 2). These chains are further linked by C—H···π interactions. The methyl group C61 acts as a donor to the fluorinated aryl ring C41—C46 (symmetry code: -*x* + 1/2, -*y* + 3/2, -*z* + 1) and also to the pirymidine ring (-*x* + 1/2, -*y* + 1/2, -*z* + 1). Additionally, the C53—H53 group acts as a donor of C—H···π(arene) interaction to the benzene ring C21—C26 (-*x* + 1/2, -*y* + 3/2, -*z* + 1). Combination of these interactions leads to layers of molecules parallel to the *bc* plane (Fig. 3).

### Experimental

The title compound was obtained by adopting the procedure described previously by Cieplik *et al.* (2003). 4 g (0.0122 mmol) of 5-(chloromethyl)-*N*-(2-fluorophenyl)-6-methyl-2-phenylpyrimidin-4-amine was dissolved in 50 ml of chloroform, and 2 g of 4-methoxyaniline was added. The reaction mixture was refluxed for 6 h with vigorous stirring, then was cooled and poured into 100 ml of water. The aqueous solution was extracted three times with chloroform (50 ml). The combined chloroform phases were dried over MgSO_4_, filtered and concentrated under vacuum. The oily residue was purified by column chromatography on silica gel (200–400 mesh) using CHCl_3_ as the eluent and by crystallization from methanol to give single crystals (yield: 78.5%, m.p. 157–158 °C).

### Refinement

The N-bonded H atoms were found from difference Fourier maps and refined with *U*_iso_(H)=1.2*U*_eq_(N). The remaining H atoms were treated as riding on their carrier atoms, with C—H distances in the range 0.95–0.99 Å, and refined with *U*_iso_(H)=1.2*U*_eq_(C) except methyl groups where *U*_iso_(H)=1.5 *U*_eq_(C).

### Crystal data

**Table:**

C_25_H_23_FN_4_O	*F*(000) = 1744
*M**_r_* = 414.47	*D*_x_ = 1.339 Mg m^−^^3^
Monoclinic, *C*2/*c*	Mo *K*α radiation, λ = 0.71073 Å
Hall symbol: -C 2yc	Cell parameters from 14660 reflections
*a* = 27.075 (11) Å	θ = 4.2–38.5°
*b* = 8.922 (4) Å	µ = 0.09 mm^−^^1^
*c* = 22.983 (10) Å	*T* = 85 K
β = 132.22 (5)°	Block, light yellow
*V* = 4112 (3) Å^3^	0.53 × 0.17 × 0.14 mm
*Z* = 8	

### Data collection

**Table:**

Oxford Diffraction Xcalibur PX κ-geometry diffractometer	7196 reflections with *I* > 2σ(*I*)
Radiation source: normal-focus sealed tube	*R*_int_ = 0.025
graphite	θ_max_ = 38.5°, θ_min_ = 4.2°
φ and ω scans	*h* = −46→40
28521 measured reflections	*k* = −12→15
10469 independent reflections	*l* = −35→39

### Refinement

**Table:**

Refinement on *F*^2^	Primary atom site location: structure-invariant direct methods
Least-squares matrix: full	Secondary atom site location: difference Fourier map
*R*[*F*^2^ > 2σ(*F*^2^)] = 0.044	Hydrogen site location: inferred from neighbouring sites
*wR*(*F*^2^) = 0.121	H atoms treated by a mixture of independent and constrained refinement
*S* = 1.01	*w* = 1/[σ^2^(*F*_o_^2^) + (0.070*P*)^2^] where *P* = (*F*_o_^2^ + 2*F*_c_^2^)/3
10469 reflections	(Δ/σ)_max_ = 0.001
288 parameters	Δρ_max_ = 0.61 e Å^−^^3^
0 restraints	Δρ_min_ = −0.22 e Å^−^^3^

### Special details

**Table:**

Geometry. All e.s.d.'s (except the e.s.d. in the dihedral angle between two l.s. planes) are estimated using the full covariance matrix. The cell e.s.d.'s are taken into account individually in the estimation of e.s.d.'s in distances, angles and torsion angles; correlations between e.s.d.'s in cell parameters are onlyused when they are defined by crystal symmetry. An approximate (isotropic) treatment of cell e.s.d.'s is used for estimating e.s.d.'s involving l.s. planes.
Refinement. Refinement of *F*^2^ against ALL reflections. The weighted *R*-factor *wR* and goodness of fit *S* are based on *F*^2^, conventional *R*-factors *R* are based on *F*, with *F* set to zero for negative *F*^2^. The threshold expression of *F*^2^ > σ(*F*^2^) is used only for calculating *R*-factors(gt) *etc*. and is not relevant to the choice of reflections for refinement. *R*-factors based on *F*^2^ are statistically about twice as large as those based on *F*, and *R*-factors based on ALL data will be even larger.

### Fractional atomic coordinates and isotropic or equivalent isotropic displacement parameters (Å^2^)

**Table:**

	*x*	*y*	*z*	*U*_iso_*/*U*_eq_	
N1	0.23426 (3)	0.36669 (7)	0.40998 (3)	0.01317 (11)	
C2	0.17473 (4)	0.43335 (8)	0.36504 (4)	0.01210 (12)	
C21	0.12462 (4)	0.39073 (8)	0.28059 (4)	0.01287 (12)	
C22	0.14297 (4)	0.30769 (8)	0.24607 (4)	0.01447 (13)	
H22	0.1884	0.2809	0.2763	0.017*	
C23	0.09517 (4)	0.26420 (9)	0.16781 (4)	0.01767 (14)	
H23	0.1081	0.2075	0.1449	0.021*	
C24	0.02852 (4)	0.30321 (9)	0.12281 (5)	0.02034 (16)	
H24	−0.0041	0.2721	0.0695	0.024*	
C25	0.00996 (4)	0.38787 (10)	0.15623 (5)	0.02228 (16)	
H25	−0.0354	0.4157	0.1256	0.027*	
C26	0.05782 (4)	0.43180 (10)	0.23455 (4)	0.01855 (14)	
H26	0.0449	0.4904	0.2570	0.022*	
N3	0.15437 (3)	0.53341 (7)	0.38901 (3)	0.01317 (11)	
C4	0.19708 (4)	0.56283 (8)	0.46609 (4)	0.01252 (12)	
N4	0.17917 (3)	0.66198 (7)	0.49490 (4)	0.01472 (12)	
H4	0.2030 (5)	0.6531 (12)	0.5456 (6)	0.018*	
C41	0.11961 (4)	0.74116 (8)	0.45602 (4)	0.01369 (12)	
C42	0.11209 (4)	0.81165 (8)	0.50421 (4)	0.01637 (14)	
F4	0.16300 (3)	0.79604 (6)	0.58317 (3)	0.02284 (11)	
C43	0.05679 (4)	0.89387 (9)	0.47602 (5)	0.02065 (15)	
H43	0.0539	0.9390	0.5111	0.025*	
C44	0.00524 (4)	0.90943 (10)	0.39507 (5)	0.02180 (16)	
H44	−0.0338	0.9642	0.3740	0.026*	
C45	0.01153 (4)	0.84392 (10)	0.34542 (5)	0.02132 (16)	
H45	−0.0233	0.8563	0.2902	0.026*	
C46	0.06771 (4)	0.76038 (9)	0.37476 (4)	0.01719 (14)	
H46	0.0708	0.7165	0.3396	0.021*	
C5	0.26157 (4)	0.49669 (8)	0.52009 (4)	0.01261 (12)	
C57	0.30861 (4)	0.53792 (9)	0.60615 (4)	0.01576 (13)	
H571	0.3492	0.4750	0.6358	0.019*	
H572	0.3223	0.6440	0.6127	0.019*	
N5	0.27693 (3)	0.51636 (7)	0.63795 (4)	0.01484 (12)	
H5	0.2652 (5)	0.4199 (13)	0.6350 (6)	0.018*	
C51	0.30334 (4)	0.58811 (8)	0.70885 (4)	0.01285 (12)	
C52	0.33894 (4)	0.72198 (8)	0.73410 (4)	0.01505 (13)	
H52	0.3495	0.7634	0.7055	0.018*	
C53	0.35944 (4)	0.79668 (8)	0.80092 (4)	0.01564 (13)	
H53	0.3834	0.8882	0.8171	0.019*	
C54	0.34466 (4)	0.73683 (8)	0.84340 (4)	0.01471 (13)	
O5	0.36179 (3)	0.80239 (6)	0.90928 (3)	0.02145 (13)	
C58	0.39084 (5)	0.94760 (9)	0.92941 (5)	0.02204 (16)	
H581	0.4332	0.9422	0.9417	0.033*	
H582	0.3990	0.9848	0.9753	0.033*	
H583	0.3604	1.0159	0.8850	0.033*	
C55	0.30912 (4)	0.60230 (8)	0.81897 (4)	0.01540 (13)	
H55	0.2988	0.5610	0.8478	0.018*	
C56	0.28893 (4)	0.52895 (8)	0.75279 (4)	0.01431 (13)	
H56	0.2650	0.4374	0.7369	0.017*	
C6	0.27830 (4)	0.40137 (8)	0.48783 (4)	0.01261 (12)	
C61	0.34603 (4)	0.33005 (9)	0.53604 (4)	0.01562 (13)	
H611	0.3474	0.2675	0.5021	0.023*	
H612	0.3547	0.2678	0.5772	0.023*	
H613	0.3801	0.4084	0.5602	0.023*	

### Atomic displacement parameters (Å^2^)

**Table:**

	*U*^11^	*U*^22^	*U*^33^	*U*^12^	*U*^13^	*U*^23^
N1	0.0143 (3)	0.0145 (3)	0.0130 (2)	0.0007 (2)	0.0101 (2)	0.00032 (19)
C2	0.0143 (3)	0.0121 (3)	0.0127 (3)	0.0001 (2)	0.0103 (2)	0.0006 (2)
C21	0.0147 (3)	0.0129 (3)	0.0128 (3)	−0.0003 (2)	0.0100 (2)	0.0001 (2)
C22	0.0167 (3)	0.0139 (3)	0.0142 (3)	0.0011 (2)	0.0110 (3)	−0.0002 (2)
C23	0.0225 (4)	0.0154 (3)	0.0152 (3)	0.0009 (3)	0.0127 (3)	−0.0015 (2)
C24	0.0219 (4)	0.0185 (3)	0.0149 (3)	−0.0009 (3)	0.0101 (3)	−0.0025 (3)
C25	0.0154 (3)	0.0271 (4)	0.0175 (3)	0.0011 (3)	0.0082 (3)	−0.0034 (3)
C26	0.0158 (3)	0.0227 (4)	0.0172 (3)	0.0004 (3)	0.0111 (3)	−0.0031 (3)
N3	0.0159 (3)	0.0133 (3)	0.0130 (2)	0.0015 (2)	0.0108 (2)	0.00088 (19)
C4	0.0158 (3)	0.0115 (3)	0.0136 (3)	0.0006 (2)	0.0112 (2)	0.0008 (2)
N4	0.0171 (3)	0.0160 (3)	0.0125 (2)	0.0041 (2)	0.0105 (2)	0.0012 (2)
C41	0.0165 (3)	0.0115 (3)	0.0168 (3)	0.0010 (2)	0.0127 (3)	0.0006 (2)
C42	0.0215 (3)	0.0143 (3)	0.0186 (3)	0.0011 (3)	0.0156 (3)	0.0005 (2)
F4	0.0304 (3)	0.0232 (3)	0.0176 (2)	0.0073 (2)	0.0172 (2)	0.00160 (18)
C43	0.0261 (4)	0.0173 (3)	0.0278 (4)	0.0026 (3)	0.0219 (3)	−0.0006 (3)
C44	0.0181 (3)	0.0187 (4)	0.0292 (4)	0.0020 (3)	0.0162 (3)	−0.0018 (3)
C45	0.0167 (3)	0.0206 (4)	0.0214 (3)	0.0021 (3)	0.0106 (3)	−0.0014 (3)
C46	0.0166 (3)	0.0177 (3)	0.0170 (3)	0.0017 (3)	0.0112 (3)	−0.0008 (2)
C5	0.0141 (3)	0.0130 (3)	0.0123 (3)	−0.0001 (2)	0.0094 (2)	0.0003 (2)
C57	0.0149 (3)	0.0204 (3)	0.0134 (3)	−0.0007 (3)	0.0100 (3)	−0.0023 (2)
N5	0.0194 (3)	0.0149 (3)	0.0129 (2)	−0.0030 (2)	0.0120 (2)	−0.0010 (2)
C51	0.0133 (3)	0.0143 (3)	0.0114 (3)	0.0012 (2)	0.0085 (2)	0.0012 (2)
C52	0.0196 (3)	0.0153 (3)	0.0144 (3)	−0.0015 (3)	0.0131 (3)	0.0006 (2)
C53	0.0201 (3)	0.0143 (3)	0.0156 (3)	−0.0019 (3)	0.0132 (3)	−0.0004 (2)
C54	0.0194 (3)	0.0143 (3)	0.0143 (3)	0.0017 (2)	0.0129 (3)	0.0012 (2)
O5	0.0370 (3)	0.0165 (3)	0.0200 (3)	−0.0041 (2)	0.0229 (3)	−0.0029 (2)
C58	0.0306 (4)	0.0176 (4)	0.0187 (3)	−0.0027 (3)	0.0169 (3)	−0.0031 (3)
C55	0.0204 (3)	0.0145 (3)	0.0173 (3)	0.0018 (3)	0.0151 (3)	0.0025 (2)
C56	0.0165 (3)	0.0138 (3)	0.0163 (3)	−0.0007 (2)	0.0125 (3)	0.0009 (2)
C6	0.0142 (3)	0.0128 (3)	0.0134 (3)	0.0001 (2)	0.0103 (2)	0.0010 (2)
C61	0.0153 (3)	0.0176 (3)	0.0154 (3)	0.0025 (3)	0.0109 (3)	0.0014 (2)

### Geometric parameters (Å, °)

**Table:**

N1—C2	1.3345 (13)	C45—H45	0.9500
N1—C6	1.3607 (15)	C46—H46	0.9500
C2—N3	1.3461 (10)	C5—C6	1.3890 (11)
C2—C21	1.4878 (16)	C5—C57	1.5112 (16)
C21—C26	1.3974 (15)	C57—N5	1.4661 (11)
C21—C22	1.3984 (11)	C57—H571	0.9900
C22—C23	1.3899 (16)	C57—H572	0.9900
C22—H22	0.9500	N5—C51	1.4178 (12)
C23—C24	1.3925 (16)	N5—H5	0.904 (11)
C23—H23	0.9500	C51—C52	1.3931 (12)
C24—C25	1.3900 (13)	C51—C56	1.4073 (11)
C24—H24	0.9500	C52—C53	1.4019 (12)
C25—C26	1.3921 (16)	C52—H52	0.9500
C25—H25	0.9500	C53—C54	1.3868 (11)
C26—H26	0.9500	C53—H53	0.9500
N3—C4	1.3384 (14)	C54—O5	1.3807 (11)
C4—N4	1.3739 (10)	C54—C55	1.3985 (12)
C4—C5	1.4230 (15)	O5—C58	1.4220 (12)
N4—C41	1.4004 (13)	C58—H581	0.9800
N4—H4	0.879 (11)	C58—H582	0.9800
C41—C46	1.4018 (17)	C58—H583	0.9800
C41—C42	1.4024 (11)	C55—C56	1.3867 (12)
C42—F4	1.3605 (16)	C55—H55	0.9500
C42—C43	1.3770 (13)	C56—H56	0.9500
C43—C44	1.3924 (18)	C6—C61	1.5050 (15)
C43—H43	0.9500	C61—H611	0.9800
C44—C45	1.3905 (13)	C61—H612	0.9800
C44—H44	0.9500	C61—H613	0.9800
C45—C46	1.3948 (13)		
			
C2—N1—C6	116.86 (8)	C6—C5—C4	115.94 (8)
N1—C2—N3	126.33 (7)	C6—C5—C57	123.87 (7)
N1—C2—C21	117.65 (8)	C4—C5—C57	120.10 (8)
N3—C2—C21	115.98 (7)	N5—C57—C5	111.08 (8)
C26—C21—C22	118.80 (8)	N5—C57—H571	109.4
C26—C21—C2	120.16 (9)	C5—C57—H571	109.4
C22—C21—C2	121.03 (8)	N5—C57—H572	109.4
C23—C22—C21	120.35 (8)	C5—C57—H572	109.4
C23—C22—H22	119.8	H571—C57—H572	108.0
C21—C22—H22	119.8	C51—N5—C57	120.10 (7)
C22—C23—C24	120.43 (9)	C51—N5—H5	113.4 (6)
C22—C23—H23	119.8	C57—N5—H5	112.4 (7)
C24—C23—H23	119.8	C52—C51—C56	118.06 (7)
C25—C24—C23	119.65 (8)	C52—C51—N5	122.64 (7)
C25—C24—H24	120.2	C56—C51—N5	119.11 (7)
C23—C24—H24	120.2	C51—C52—C53	121.18 (7)
C24—C25—C26	119.96 (8)	C51—C52—H52	119.4
C24—C25—H25	120.0	C53—C52—H52	119.4
C26—C25—H25	120.0	C54—C53—C52	119.94 (8)
C25—C26—C21	120.79 (9)	C54—C53—H53	120.0
C25—C26—H26	119.6	C52—C53—H53	120.0
C21—C26—H26	119.6	O5—C54—C53	124.45 (7)
C4—N3—C2	116.40 (7)	O5—C54—C55	115.93 (7)
N3—C4—N4	119.57 (7)	C53—C54—C55	119.61 (7)
N3—C4—C5	122.37 (8)	C54—O5—C58	116.58 (6)
N4—C4—C5	118.05 (7)	O5—C58—H581	109.5
C4—N4—C41	130.19 (7)	O5—C58—H582	109.5
C4—N4—H4	113.6 (7)	H581—C58—H582	109.5
C41—N4—H4	113.3 (7)	O5—C58—H583	109.5
N4—C41—C46	127.26 (8)	H581—C58—H583	109.5
N4—C41—C42	116.00 (8)	H582—C58—H583	109.5
C46—C41—C42	116.73 (8)	C56—C55—C54	120.20 (7)
F4—C42—C43	119.27 (8)	C56—C55—H55	119.9
F4—C42—C41	116.93 (8)	C54—C55—H55	119.9
C43—C42—C41	123.80 (9)	C55—C56—C51	121.01 (8)
C42—C43—C44	118.59 (9)	C55—C56—H56	119.5
C42—C43—H43	120.7	C51—C56—H56	119.5
C44—C43—H43	120.7	N1—C6—C5	121.95 (7)
C45—C44—C43	119.27 (8)	N1—C6—C61	115.44 (8)
C45—C44—H44	120.4	C5—C6—C61	122.62 (8)
C43—C44—H44	120.4	C6—C61—H611	109.5
C44—C45—C46	121.54 (9)	C6—C61—H612	109.5
C44—C45—H45	119.2	H611—C61—H612	109.5
C46—C45—H45	119.2	C6—C61—H613	109.5
C45—C46—C41	120.04 (8)	H611—C61—H613	109.5
C45—C46—H46	120.0	H612—C61—H613	109.5
C41—C46—H46	120.0		
			
C6—N1—C2—N3	1.13 (11)	C44—C45—C46—C41	−0.17 (13)
C6—N1—C2—C21	−176.79 (6)	N4—C41—C46—C45	−179.60 (8)
N1—C2—C21—C26	166.64 (7)	C42—C41—C46—C45	−1.20 (11)
N3—C2—C21—C26	−11.50 (10)	N3—C4—C5—C6	1.19 (10)
N1—C2—C21—C22	−12.51 (10)	N4—C4—C5—C6	−177.79 (6)
N3—C2—C21—C22	169.35 (7)	N3—C4—C5—C57	177.97 (7)
C26—C21—C22—C23	−1.44 (11)	N4—C4—C5—C57	−1.00 (10)
C2—C21—C22—C23	177.72 (7)	C6—C5—C57—N5	−129.73 (8)
C21—C22—C23—C24	0.23 (12)	C4—C5—C57—N5	53.76 (10)
C22—C23—C24—C25	0.84 (12)	C5—C57—N5—C51	−161.21 (6)
C23—C24—C25—C26	−0.68 (13)	C57—N5—C51—C52	27.18 (11)
C24—C25—C26—C21	−0.55 (13)	C57—N5—C51—C56	−157.88 (7)
C22—C21—C26—C25	1.60 (12)	C56—C51—C52—C53	−0.53 (11)
C2—C21—C26—C25	−177.57 (7)	N5—C51—C52—C53	174.46 (7)
N1—C2—N3—C4	−3.45 (11)	C51—C52—C53—C54	0.40 (12)
C21—C2—N3—C4	174.50 (6)	C52—C53—C54—O5	−179.13 (7)
C2—N3—C4—N4	−178.93 (6)	C52—C53—C54—C55	−0.22 (12)
C2—N3—C4—C5	2.12 (10)	C53—C54—O5—C58	6.20 (11)
N3—C4—N4—C41	1.78 (12)	C55—C54—O5—C58	−172.75 (7)
C5—C4—N4—C41	−179.22 (7)	O5—C54—C55—C56	179.18 (7)
C4—N4—C41—C46	−13.42 (13)	C53—C54—C55—C56	0.17 (12)
C4—N4—C41—C42	168.17 (7)	C54—C55—C56—C51	−0.31 (11)
N4—C41—C42—F4	−0.01 (10)	C52—C51—C56—C55	0.48 (11)
C46—C41—C42—F4	−178.59 (7)	N5—C51—C56—C55	−174.69 (7)
N4—C41—C42—C43	−179.89 (8)	C2—N1—C6—C5	2.61 (10)
C46—C41—C42—C43	1.53 (12)	C2—N1—C6—C61	−177.14 (6)
F4—C42—C43—C44	179.69 (7)	C4—C5—C6—N1	−3.64 (10)
C41—C42—C43—C44	−0.43 (13)	C57—C5—C6—N1	179.71 (7)
C42—C43—C44—C45	−1.00 (13)	C4—C5—C6—C61	176.09 (7)
C43—C44—C45—C46	1.31 (13)	C57—C5—C6—C61	−0.56 (11)

### Hydrogen-bond geometry (Å, °)

**Table:**

Cg1, Cg2 and Cg3 are the centroids of the N1–C6, C41–C46 and C21–C26 rings, respectively.

**Table:**

*D*—H···*A*	*D*—H	H···*A*	*D*···*A*	*D*—H···*A*
N4—H4···N5	0.88 (1)	2.07 (1)	2.794 (2)	139 (1)
N4—H4···F4	0.88 (1)	2.19 (1)	2.633 (1)	111 (1)
C61—H611···O5^i^	0.98	2.49	3.426 (2)	159
C61—H612···Cg1^ii^	0.98	2.69	3.381 (3)	128
C61—H613···Cg2^iii^	0.98	2.73	3.657 (3)	159
C53—H53···Cg3^ii^	0.95	2.74	3.634 (3)	156
N5—H5···N1^iii^	0.90 (1)	2.76 (1)	3.541 (2)	145 (1)

Symmetry codes: (i) *x*, −*y*+1, *z*−1/2; (ii) −*x*+1/2, −*y*+3/2, −*z*+1; (iii) −*x*+1/2, −*y*+1/2, −*z*+1.
